# Lower serum chloride concentrations are associated with increased risk of mortality in critically ill cirrhotic patients: an analysis of the MIMIC-III database

**DOI:** 10.1186/s12876-021-01797-3

**Published:** 2021-05-01

**Authors:** Yun Ji, Libin Li

**Affiliations:** Department of Surgical Intensive Care Unit, The Second Affiliated Hospital, School of Medicine, Zhejiang University, 88 Jiefang Road, Hangzhou, 310009 Zhejiang China

**Keywords:** Hypochloremia, Liver cirrhosis, Mortality, Critical illness

## Abstract

**Background:**

Cirrhosis can be complicated by electrolyte abnormalities, but the major focus has been concentrated on the clinical significance of serum sodium levels. Emerging studies have identified hypochloremia as an independent prognostic marker in patients with chronic heart failure and chronic kidney disease. The aim of this study was to investigate whether serum chloride levels were associated with mortality of critically ill cirrhotic patients.

**Methods:**

Critically ill cirrhotic patients were identified from the Multi-parameter Intelligent Monitoring in Intensive Care III Database. The primary outcome was ICU mortality. Logistic regression was used to explore the association between serum chloride levels and ICU mortality. The area under the receiver operating characteristic curves (AUC) was used to assess the performance of serum chloride levels for predicting ICU mortality.

**Results:**

A total of 1216 critically ill cirrhotic patients were enrolled in this study. The overall ICU mortality rate was 18.8%. Patients with hypochloremia had a higher ICU mortality than those with non-hypochloremia (34.2% vs. 15.8%; *p* < 0.001). After multivariable risk adjustment for age, gender, ethnicity, chloride, sodium, Model for End-stage Liver Disease score, Sequential Organ Failure Assessment score, Elixhauser comorbidity index, mechanical ventilation, vasopressors, renal replacement therapy, acute kidney injury, hemoglobin, platelet, and white blood cell, serum chloride levels remained independently associated with ICU mortality (OR 0.94; 95% CI 0.91–0.98; *p* = 0.002) in contrast to serum sodium levels, which were no longer significant (OR 1.03; 95% CI 0.99–1.08; *p* = 0.119). The AUC of serum chloride levels (AUC, 0.600; 95% CI 0.556–0.643) for ICU mortality was statistically higher than that of serum sodium levels (AUC, 0.544; 95% CI 0.499–0.590) (*p* < 0.001).

**Conclusions:**

In critically ill cirrhotic patients, serum chloride levels are independently and inversely associated with ICU mortality, thus highlighting the prognostic role of serum chloride levels which are largely overlooked.

## Background

Abnormalities in serum electrolyte levels often complicate cirrhosis [1]. Serum sodium has long been considered as an important electrolyte prognostic marker [2, 3]. It has been suggested that the addition of serum sodium to the Model for End-stage Liver Disease (MELD) score would increase its accuracy in predicting mortality [4]. However, the prognostic value of serum chloride and its interplay with serum sodium in patients with cirrhosis is less well understood despite its broad availability in routinely used blood chemistry panels.

Chloride as the principal extracellular anion accounts for approximately one-third of plasma tonicity and two-thirds of negative charges [5]. Serum chloride performs a large number of functions in the body including the maintenance of osmotic pressure, acid–base balance, muscular activity, and the regulation of body fluid distribution [6]. Emerging studies have identified hypochloremia as an independent prognostic marker in patients with chronic heart failure and chronic kidney disease [7–12]. However, serum chloride has scarcely been investigated as a potential biomarker of cirrhosis. Therefore, we aimed to investigate whether serum chloride levels were associated with mortality of critically ill cirrhotic patients.

## Methods

### Data source

All data in this study were extracted from the Multiparameter Intelligent Monitoring in Intensive Care (MIMIC) III database version 1.4 [13]. MIMIC-III is an openly available database developed by the computational physiology laboratory of Massachusetts Institute of Technology. The database contains de-identified clinical data for over 50,000 adult intensive care unit (ICU) stays at Beth Israel Deaconess Medical Center in Boston, MA, from 2001 to 2012. The institutional review boards of the Massachusetts Institute of Technology and Beth Israel Deaconess Medical Center approved the establishment and use of the database.

### Study population and variable extraction

The primary study population consists of adult (age ≥ 18 years) ICU patients with cirrhosis. We defined hypochloremia as a serum chloride level less than 99 mEq/L. We defined hyponatremia as a serum sodium level less than 135 mEq/L. We excluded patients with: (i) liver transplantation, (ii) chronic renal failure which was defined by the Elixhauser comorbidity index, and (iii) initial chloride measurements completed more than 24 h after ICU admission. For patients who were admitted to the ICU more than once, only the first ICU stay was considered in this study. Records containing baseline characteristics were extracted within the first 24 h after admission.

Baseline variables included patient demographics (eg, age, gender), etiology of cirrhosis, MELD score, Sequential Organ Failure Assessment (SOFA) score, and measures of organ support (eg, mechanical ventilation, vasopressors, renal replacement therapy). The MELD score was calculated according to the policy of the Organ Procurement and Transplant Network (OPTN) [14]. Laboratory data including serum chloride and sodium values, international normalized ratio, bilirubin, creatinine, hemoglobin, platelet count, and white blood cell count were measured during the first 24 h in the ICU. If a variable was recorded several times in the first 24 h, we used the first value to analyze. A 1.5-fold increase in the admission serum creatinine level relative to the baseline was considered to reflect acute kidney injury (AKI) according to the Kidney Disease Improving Global Outcome criteria [15]. The minimum value of serum creatinine available within the 7 days before admission was used as the baseline serum creatinine level. For patients without previous serum creatinine, it was estimated using the following formula: serum creatinine level = 0.74—0.2 (if female) + 0.08 (if black) + 0.003 × age (in years) [16]. The primary outcome of interest was ICU mortality, defined as death before ICU discharge.

### Statistical analysis

Categorical data were shown as frequency (percent), while continuous ones as mean (standard deviation [SD]) or median (interquartile range [IQR]). We did comparisons between groups by the χ^2^ test or Fisher’s exact test for categorical data and the Student t test or the Wilcoxon rank-sum test for continuous ones. A multivariable logistic model was constructed to evaluate the association between the occurrence of hypochloremia and mortality. Covariables used in the multivariable logistic model were age, gender, ethnicity, MELD score, SOFA score, Elixhauser comorbidity index, mechanical ventilation, vasopressors, renal replacement therapy, AKI, hemoglobin, platelet, white blood cell, and sodium. These covariables were selected based on clinical relevance for risk of death (age, gender) or statistical criteria (univariable *p* < 0.05 for inclusion in the analysis). International normalized ratio, bilirubin and creatinine are included in the MELD score and were therefore not separately included in the multivariable logistic model. The results were expressed as odds ratios (ORs) with 95% confidence intervals (CIs). The authors expected that the effect of hypochloremia on mortality would vary depending on patient characteristics. Accordingly, this potential heterogeneity was assessed by subgroup analyses stratified by age, gender, ethnicity, ICU type, etiology of cirrhosis, Elixhauser comorbidity index, MELD score, SOFA score, international normalized ratio, bilirubin, creatinine, hemoglobin, platelet, white blood cell, vasopressors, and mechanical ventilation. Cut-off values of continuous data for subgroup analyses were based on the median value of the whole study population. The Lowess smoothing technique was further used to explore the relationship between chloride and mortality [17]. The area under the receiver operating characteristic curves (AUC) was used to assess the performance of chloride and sodium for predicting mortality. Bonferroni correction was used for multiple comparisons, if needed. A two-tailed test was performed, and* p* < 0.05 was considered statistically significant. All statistical analyses were performed using STATA V.16.0.

## Results

### Baseline characteristics

**Fig. 1:**
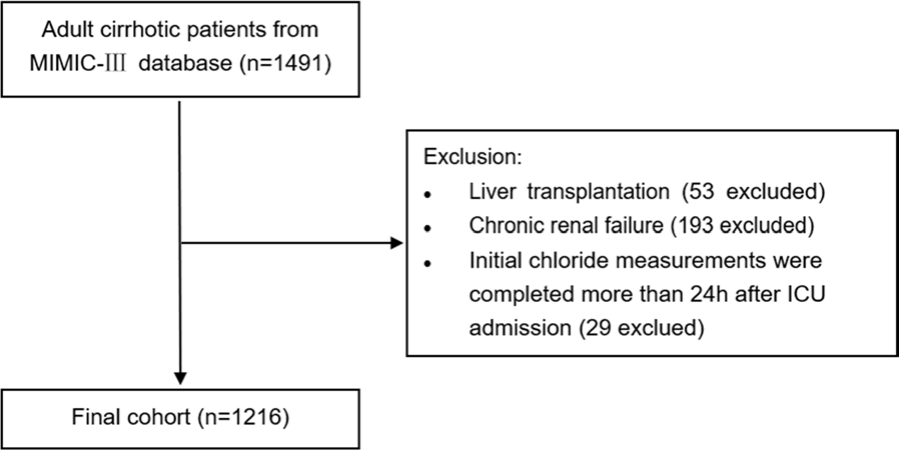
Flow chart of patient selection

Data on 1216 patients were included in this study (Fig. 1). The baseline characteristics of the cohort are listed in Table 1. The overall median age was 56 years, 67.5% were men, 69.5% were white, and 48.8% were alcohol-related. The median Elixhauser comorbidity index was 19, the median MELD score was 18, and the median SOFA score was 7. Mechanical ventilation, vasopressors, and renal replacement therapy were required for 46.7%, 25.6%, and 4.1% of patients, respectively.

**Table 1:** Baseline characteristics of study participants

	Total patients (n = 1216)	Chloride < 99 mEq/L (n = 199)	Chloride ≥ 99 mEq/L (n = 1017)	*p* value
Age, years	56 (49–65)	54 (49–63)	57 (50–65)	0.056
Male, n (%)	821 (67.5)	129 (64.8)	692 (68.0)	0.375
Ethnicity, n (%)				0.087
White	845 (69.5)	150 (75.4)	695 (68.3)	
Black	88 (7.2)	8 (4.0)	80 (7.9)	
Hispanic	69 (5.7)	7 (3.5)	62 (6.1)	
Other	214 (17.6)	34 (17.1)	180 (17.7)	
ICU type, n (%)				0.157
MICU	741 (60.9)	130 (65.3)	611 (60.1)	
SICU/TSICU	362 (29.8)	48 (24.1)	314 (30.9)	
CCU/CSRU	113 (9.3)	21 (10.6)	92 (9.1)	
Etiology of cirrhosis, n (%)				0.165
Alcoholic	593 (48.8)	106 (53.3)	487 (47.9)	
Non-alcoholic	623 (51.2)	93 (46.7)	530 (52.1)	
Elixhauser comorbidity index	19 (12–30)	25 (17–32)	19 (11–29)	< 0.001
MELD score	18 (13–27)	26 (18–38)	17 (13–25)	< 0.001
SOFA score	7 (4–10)	8 (6–12)	6 (4–9)	< 0.001
Mechanical ventilation, n (%)	568 (46.7)	81 (40.7)	487 (47.9)	0.063
Vasopressors, n (%)	311 (25.6)	62 (31.2)	249 (24.5)	0.049
Renal replacement therapy, n (%)	50 (4.1)	17 (8.5)	33 (3.2)	0.001
Acute kidney injury, n (%)	240 (19.7)	79 (39.7)	161 (15.8)	< 0.001
Blood tests results				
Chloride, mEq/L	105 (101–109)	95 (92–97)	107 (103–110)	< 0.001
Sodium, mEq/L	137 (134–141)	129 (125–133)	139 (136–141)	< 0.001
Bilirubin, mg/dL	3.0 (1.5–7.1)	6.2 (2.1–17.0)	2.8 (1.4–5.6)	< 0.001
Creatinine, mg/dL	1.0 (0.7–1.5)	1.4 (0.9–2.7)	0.9 (0.7–1.4)	< 0.001
International normalized ratio	1.6 (1.4–2.0)	1.9 (1.5–2.7)	1.6 (1.3–1.9)	< 0.001
Hemoglobin, g/dL	10.2 (9.0–11.5)	10.2 (8.9–11.7)	10.2 (9.0–11.5)	0.769
Platelet, 10^9^/L	106 (71–157)	106 (71–156)	106 (71–157)	0.986
White blood cell, 10^9^/L	8.7 (5.5–13.6)	9.9 (7.1–15.8)	8.4 (5.3–13.0)	< 0.001
Outcome				
ICU mortality, n (%)	229 (18.8)	68 (34.2)	161 (15.8)	< 0.001

CCU, coronary care unit; CSRU, cardiac surgery care unit; ICU, intensive care unit; MELD, Model for End-stage Liver Disease; MICU, medical intensive care unit; SICU, surgical intensive care unit; SOFA, Sequential Organ Failure Assessment; TSICU, traumatic surgical intensive care unit

At baseline 199 patients (16.4%) showed serum chloride levels < 99 mEq/L. As shown in Table 1, patients with hypochloremia, when compared to those without, had lower serum sodium levels, and higher bilirubin, creatinine, international normalized ratio, and white blood cell count. The hypochloremic group had higher Elixhauser comorbidity indices, MELD scores, and SOFA scores calculated at ICU admission.

### Hypochloremia and ICU mortality

**Fig. 2:**
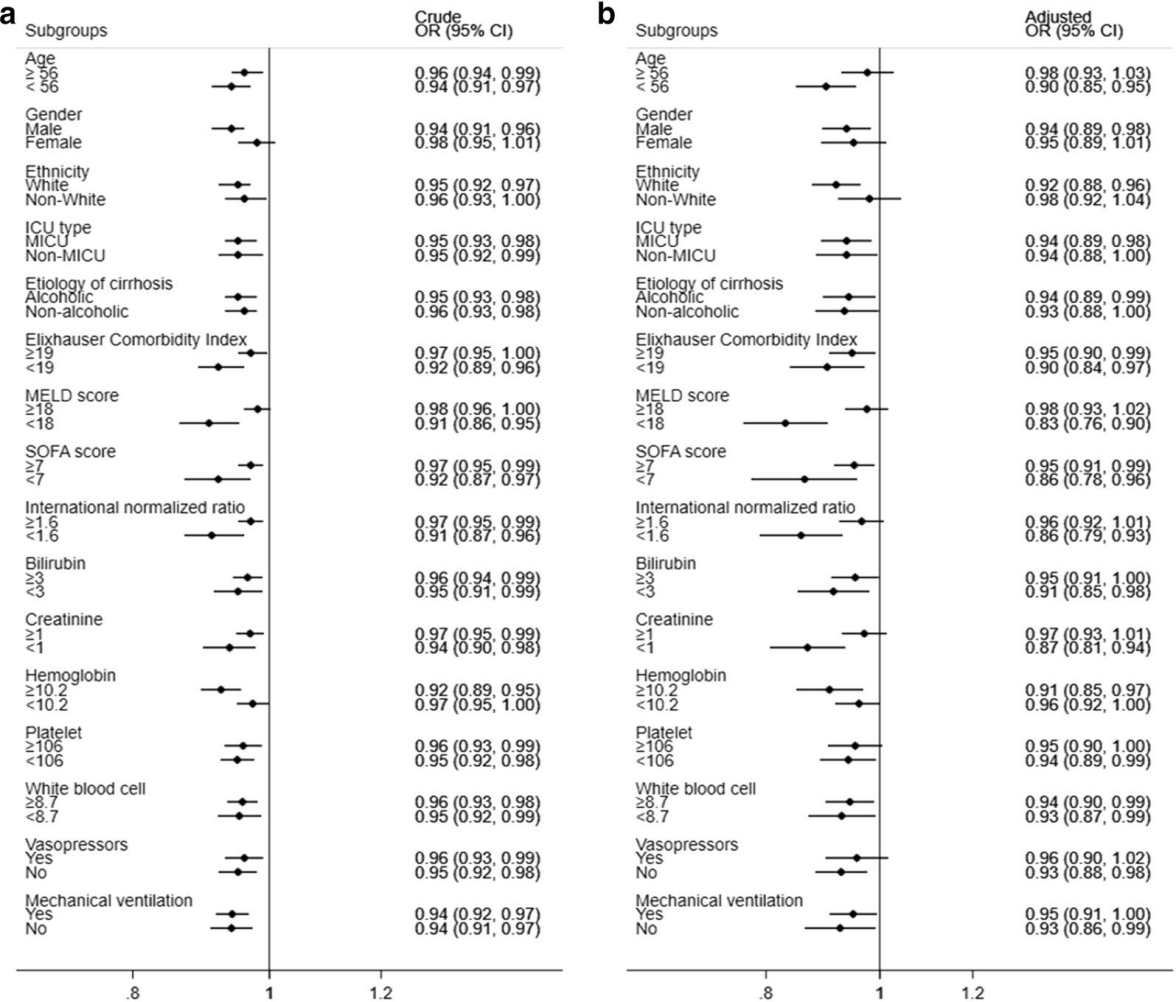
Subgroup analyses of the association between chloride and ICU mortality

The overall ICU mortality rate was 18.8%. Patients with hypochloremia were noted to have a higher ICU mortality rate of 34.2%, compared with 15.8% in the non-hypochloremic group (*p* < 0.001). Univariable analysis showed that admission chloride levels expressed as continuous variables were inversely associated with ICU mortality (OR 0.96; 95% CI 0.94–0.97; p < 0.001) (Table 2). After multivariable risk adjustment for age, gender, ethnicity, MELD score, SOFA score, Elixhauser comorbidity index, mechanical ventilation, vasopressors, renal replacement therapy, AKI, hemoglobin, platelet, white blood cell, and sodium, admission chloride levels remained independently associated with ICU mortality. Every unit decrease in chloride level was associated with a 6% relative increment in ICU mortality risk (OR 0.94; 95% CI 0.91–0.98; *p* = 0.002) (Table 2). In the subgroup analyses, the association between admission chloride levels and the risk of ICU mortality remained relatively consistent across dichotomized subgroups of: medical ICU (MICU) or non-MICU patients, alcoholic or non-alcoholic cirrhosis, Elixhauser comorbidity index less than or greater than or equal to 19, SOFA score less than or greater than or equal to 7, bilirubin less than or greater than or equal to 3 mg/dL, white blood cell count less than or greater than or equal to 8.7 × 10^9^/L, and use of mechanical ventilation (Fig. 2).

**Table 2:** Logistic regression analysis for ICU mortality

	Univariable	*p* value	Multivariable	*p* value
Chloride, per 1 mEq/L increase	0.96 (0.94–0.97)	< 0.001	0.94 (0.91–0.98)	0.002
Sodium, per 1 mEq/L increase	0.97 (0.95–0.99)	0.010	1.03 (0.99–1.08)	0.119
Age per year increase	1.01 (1.00–1.02)	0.098	1.02 (1.01–1.04)	0.002
Gender				
Female	1 [Reference]		1 [Reference]	
Male	0.83 (0.62–1.13)	0.234	0.86 (0.60–1.24)	0.422
Ethnicity				
Other	1 [Reference]		1 [Reference]	
White	0.52 (0.37–0.73)	< 0.001	0.73 (0.47–1.13)	0.155
Black	0.64 (0.36–1.17)	0.150	1.03 (0.49–2.19)	0.935
Hispanic	0.20 (0.08–0.51)	0.001	0.40 (0.13–1.18)	0.098
ICU type				
MICU	1 [Reference]			
SICU/TSICU	0.89 (0.64–1.23)	0.481		
CCU/CSRU	1.14 (0.70–1.85)	0.604		
Etiology of cirrhosis				
Non-alcoholic	1 [Reference]			
Alcoholic	1.28 (0.96–1.70)	0.097		
Elixhauser comorbidity index, per point increase	1.03 (1.02–1.04)	< 0.001	1.01 (0.99–1.02)	0.243
MELD score, per point increase	1.10 (1.09–1.12)	< 0.001	1.04 (1.01–1.06)	0.007
SOFA score, per point increase	1.39 (1.32–1.45)	< 0.001	1.20 (1.12–1.30)	< 0.001
Mechanical ventilation				
No	1 [Reference]		1 [Reference]	
Yes	3.29 (2.4–4.48)	< 0.001	2.61 (1.74–3.90)	< 0.001
Vasopressors				
No	1 [Reference]		1 [Reference]	
Yes	4.81 (3.55–6.53)	< 0.001	1.42 (0.93–2.17)	0.103
Renal replacement therapy				
No	1 [Reference]		1 [Reference]	
Yes	5.60 (3.15–9.97)	< 0.001	0.63 (0.30–1.32)	0.221
Acute kidney injury				
No	1 [Reference]		1 [Reference]	
Yes	3.81 (2.77–5.23)	< 0.001	1.56 (1.03–2.37)	0.037
Hemoglobin, per 1 g/dL increase	0.90 (0.83–0.97)	0.005	0.94 (0.86–1.03)	0.189
Platelet, per 1 × 10^9^/L increase	1.00 (1.00–1.00)	0.004	1.00 (1.00–1.00)	0.473
White blood cell, per 1 × 10^9^/L increase	1.05 (1.03–1.07)	< 0.001	1.04 (1.01–1.06)	0.008

CCU, coronary care unit; CSRU, cardiac surgery care unit; ICU, intensive care unit; MELD, Model for End-stage Liver Disease; MICU, medical intensive care unit; SICU, surgical intensive care unit; SOFA, Sequential Organ Failure Assessment; TSICU, traumatic surgical intensive care unit

### Chloride and sodium serum levels

**Fig. 3:**
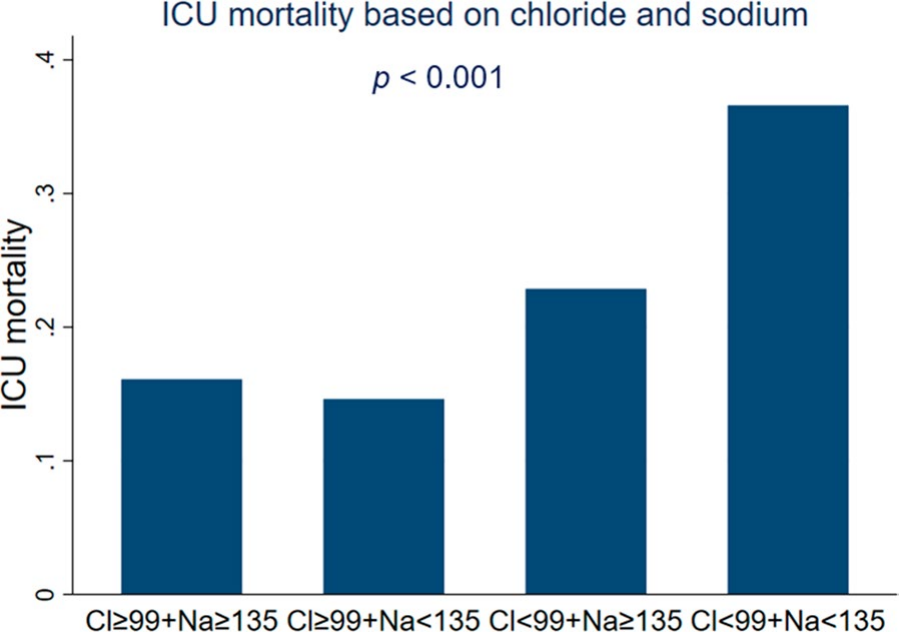
ICU mortality stratified by both serum chloride and serum sodium levels. Patients were divided into groups with neither hypochloremia nor hyponatremia, with hyponatremia alone, with hypochloremia alone, and with hypochloremia combined with hyponatremia, which were significantly different (*p* < 0.001). Pairwise comparisons with adjustment for multiple comparisons demonstrated significant differences between patients with hypochloremia combined with hyponatremia (36.6%) vs. patients with neither hypochloremia nor hyponatremia (16.1%) (*p* < 0.001) and between patients with hypochloremia combined with hyponatremia (36.6%) vs. patients with hyponatremia alone (14.6%) (*p* < 0.001)

Admission chloride and sodium levels were strongly correlated each other (r = 0.771; *p* < 0.001). ICU mortality rates of patients stratified by both serum chloride and sodium are shown in Fig. 3, which illustrates different ICU mortality rates among groups (*p* < 0.001). Pairwise comparisons between groups revealed significant differences between patients with hypochloremia combined with hyponatremia (36.6%) vs. patients with neither hypochloremia nor hyponatremia (16.1%) (*p* < 0.001) and between patients with hypochloremia combined with hyponatremia (36.6%) vs. patients with hyponatremia alone (14.6%) (*p* < 0.001) (Fig. 3).

**Fig. 4:**
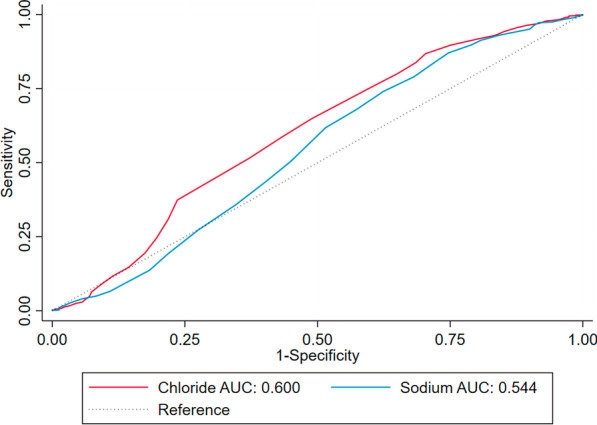
Comparison of the receiver operating characteristic curves of sodium and chloride to predict ICU mortality

At univariable analysis, like admission chloride levels, admission sodium levels were inversely associated with ICU mortality (OR 0.97; 95% CI 0.95–0.99; *p* = 0.010). However, admission sodium levels were no longer negatively associated with mortality after multivariable adjustment (OR 1.03; 95% CI 0.99–1.08; *p* = 0.119) (Table 2). The AUC for predicting ICU mortality of admission chloride levels (AUC, 0.600; 95% CI 0.556–0.643) was statistically higher than that of admission sodium levels (AUC, 0.544; 95% CI 0.499–0.590) (*p* < 0.001) (Fig. 4). Adding admission chloride levels to MELD score numerically improved the AUC from 0.770 to 0.774 for predicting ICU mortality, but this difference was not statistically significant (*p* = 0.066).

### ICU mortality according to three chloride groups

**Fig. 5:**
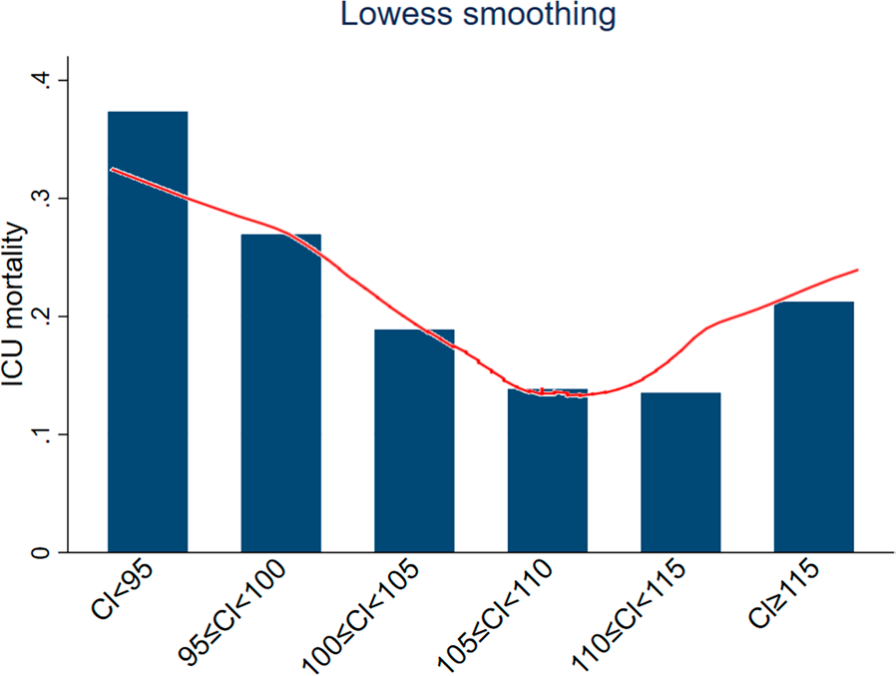
Association between chloride and ICU mortality determined using the Lowess smoothing technique

In addition to the above analyses based on 2 groups (hypochloremia vs. non-hypochloremia), patients were also divided into 3 groups: hypochloremia (< 99 mEq/L, n = 199), normochloremia (≥ 99 and < 110 mEq/L, n = 730), and hyperchloremia (≥ 110 mEq/L, n = 287). Patients with hypochloremia had a higher ICU mortality rate of 34.2%, compared with 15.9% in the normonatremic group (*p* < 0.001). However, the ICU mortality rate was similar between patients with hyperchloremia (15.7%) and with normochloremia (15.9%). Figure 5 shows the relationship between admission chloride levels and ICU mortality determined using the Lowess smoothing technique. The ICU mortality rate was numerically higher in patients with severe hyperchloremia (≥ 115 mEq/L) than in patients with normonatremia, but this difference was not statistically significant (21.3% vs. 15.9%, *p* = 0.219).

## Discussion

In this cohort study, we report several key observations regarding the prognostic importance of serum chloride in critically ill patients with cirrhosis. First, serum chloride levels were independently and inversely associated with ICU mortality, both univariately and multivariately. This result remained relatively consistent across different subgroups. Second, the ICU mortality rate was significantly higher in patients with hypochloremia, irrespective of their admission sodium levels. Third, serum sodium levels were no longer negatively associated with mortality at multivariable analysis. These findings highlight that while the link between hyponatremia and mortality has been focused on to date, we should now recognize the prognostic value of serum chloride in patients with cirrhosis.

These findings uncover two possible scenarios. In a first one, the onset of hypochloremia is the consequence of the progressive course of cirrhosis. In this case, hypochloremia is an important biomarker of cirrhosis progression. However, it is also possible that hypochloremia could mediate the worse prognosis of cirrhotic patients. In this second possible scenario, hypochloremia represents an exacerbation factor in cirrhotic patients. The observational nature of this study did not allow us to determine this answer. More work is needed.

To date, few studies have evaluated the prognostic significance of serum chloride in patients with cirrhosis [18, 19]. In decompensated cirrhotic patients in the ICU (n = 389), Sumarsono et al. [18] found that serum chloride levels were independently and inversely associated with 180-day mortality. In cirrhotic patients with acute upper gastrointestinal bleeding (n = 683), Ning et al. [19] found that the in-hospital mortality rate was 9.8% in hypochloremic group, 2.5% in normochloremic group, and 8.5% in hyperchloremic group. They also found that higher serum chloride levels were positively associated with increased in-hospital mortality of cirrhotic patients with acute upper gastrointestinal bleeding.

Our current study with 1216 enrolled patients represents the largest collective that has been analyzed for the prognostic relevance of serum chloride in cirrhotic patients to date. Our results support the findings of Sumarsono et al. [18]. However, our results were not identical to the findings of Ning et al. [19]. Both studies (ours and that of Ning et al. [19]) found that cirrhotic patients with hypochloremia had a higher mortality rate. Ning et al. [19] also found that hyperchloremia was associated with increased mortality rate, which was not identified in our study. Differences in results between the two studies might be due to different populations. In particular, we focused on ICU cirrhotic patients, whereas Ning et al. [19] focused on cirrhotic patients with acute upper gastrointestinal bleeding. Acute bleeding events, as we know, can lead to pachyemia and high blood viscosity, which may cause the electrolyte concentrations to be increased [19].

Recently, some researchers have changed their focus from sodium to its often overlooked counter ion in salt, the chloride [20]. Growing evidence have shown that hypochloremia is associated with a higher risk of mortality in patients with acute or chronic heart failure, chronic kidney disease, and pulmonary hypertension [7–12, 21]. It is noteworthy that multiple studies have shown that serum chloride had better prognostic predictability than serum sodium [9, 10, 12]. In our study, lower chloride levels were associated with higher mortality after multivariable adjustment for known prognostic factors including serum sodium. More interestingly, however, lower sodium levels were no longer associated with higher mortality when serum chloride was added to the multivariable model. Importantly, our study showed that serum chloride had a higher predictive ability for mortality when compared to serum sodium. It is therefore that serum chloride may provide more robust prognostic information than serum sodium in patients with cirrhosis. Our findings highlight the need to focus on better understanding of chloride homeostasis in patients with cirrhosis.

Whether or not a causal association exists between hypochloremia and increased mortality risk in patients with cirrhosis needs further investigation, however there are possible pathophysiological mechanisms, which might link this association. The progressive course of cirrhosis with portal hypertension and splanchnic vasodilatation leads to the development of effective arterial hypovolemia and activation of endogenous vasoconstrictor systems causing various complications, including cirrhotic cardiomyopathy, hepatorenal syndrome, and hepatopulmonary syndrome [22, 23]. Since serum chloride has been recognized to play a critical role in modulation of renin secretion, renal salt sensing, tubuloglomerular feedback, and regulation of sodium transporter [21, 24, 25], it is mechanistically conceivable that hypochloremia could promote greater cirrhosis-related complications and risk for death. Future prospective studies should examine the influence of hypochloremia on the development of cirrhosis-related complications.

In advanced cirrhosis, maladaptive neurohormonal activation (increase in arginine vasopressin and renin–angiotensin–aldosterone system) leads to increased free water retention, which may result in reduced plasma concentrations of chloride [23]. Perturbations in chloride homeostasis may be further exacerbated by frequent fluid challenges or diuretics use. Thus, we expected that fluids or diuretics given prior to admission would be a driver of admission hypochloremia. Although the impact of fluids or diuretics was not examined in our study, we think that we should pay more attention to the cirrhotic patients treated with fluids or diuretics.

The major strength of our study is the large number of patients with cirrhosis; however, there are also several limitations to our study. First, the study was limited by its retrospective nature. Second, since the study was using a relatively old database with data from 2001 to 2012, the data may not accurately reflect the current situation. Third, we evaluated chloride concentration only at one point of time in cirrhotic patients. Therefore, we were unable to assess the prognostic role of serum chloride changes in cirrhotic patients. Fourth, although we did our best to use a multivariable model to control bias, there remain the possibility of confounding variables that were not examined in our multivariable model. Fifth, we were unable to calculate the Child–Pugh score, as some of the necessary clinical data were not available in the database. Other scoring systems, such as albumin-bilirubin score [26] and chronic liver failure-sequential organ failure (CLIF-SOFA) score [27], have been recently proposed for prognostic assessment of cirrhosis. Although albumin-bilirubin score is simple, albumin was not measured in 40 percent of the study population during the first 24 h in the ICU. The CLIF-SOFA components include: SpO_2_/FiO_2_ or PaO_2_/FiO_2_, hypotension (mean arterial pressure, vasopressor use), international normalized ratio, hepatic encephalopathy grade, and serum creatinine [27]. We were unable to reliably determine hepatic encephalopathy grade in the database. Therefore, we did not assess these prognostic scores in this study. Despite these limitations, this study provides new insight into the prognostic role of chloride in cirrhotic patients. Future studies are required to assess the performance of a composite score containing serum chloride (such as MELD-Cl or MELD-Na-Cl) and to determine whether therapeutic maintenance of chloride homeostasis can improve survival in cirrhotic patients [18].

## Conclusions

In conclusion, this study shows that serum chloride levels are independently and inversely associated with ICU mortality in critically ill cirrhotic patients. This finding highlights the clinical significance of chloride, a routinely measured electrolyte, in prognostication for critically ill cirrhotic patients.

## Data Availability

The datasets presented in the current study are available in the MIMIC III database (https://physionet.org/works/MIMICIIIClinicalDatabase/files/).

## Abbreviations

CI: Confidence interval
ICU: Intensive care unit
IQR: Interquartile ranges
MELD: Model for End-stage Liver Disease
MIMIC: Medical Information Mart for Intensive Care
OR: Odds ratio
SD: Standard deviation
SOFA: Sequential organ failure assessment
